# Severe sclerosing cholangitis after Langerhans cell histiocytosis treated by liver transplantation: An adult case report

**DOI:** 10.1097/MD.0000000000005994

**Published:** 2017-03-03

**Authors:** Yunhua Tang, Zhiheng Zhang, Maogen Chen, Weiqiang Ju, Dongping Wang, Fei Ji, Qingqi Ren, Zhiyong Guo, Xiaoshun He

**Affiliations:** Organ Transplant Center, The First Affiliated Hospital of Sun Yat-sen University, Guangzhou, China.

**Keywords:** Langerhans cell histiocytosis, liver transplantation, sclerosing cholangitis

## Abstract

**Background:**

Langerhans cell histiocytosis (LCH) is a rare hematopoietic disorder of unknown pathogenesis. LCH diseases may occur in a single organ or multisystem organ. The patients with multisystem involvement usually have a poor prognosis. Liver involvement in multisystem LCH results in severe complications, such as obvious sclerosing cholangitis (SC) with jaundice.

**Methods:**

We reported a 31-year-old man developed severe SC due to multisystem LCH and was successfully treated by liver transplantation (LT). In addition, we firstly used tacrolimus and mycofenolate mofetil as immunosuppressants to treat LCH after LT.

**Results:**

We performed the immunosuppressants to deal with the LCH after LT, now the patient is currently well with normal liver function and no evidence of recurrence of LCH for 4 and a half years follow-up.

**Conclusion:**

LT should be recommended as an effective treatment for these adults with severe SC due to multisystem LCH. Finally, using tacrolimus and mycofenolate mofetil as immunosuppressants to treat LCH might be favorable to prevent LCH recurrence.

## 1 Introduction

Langerhans cell histiocytosis (LCH) is a rare hematopoietic disorder of unknown pathogenesis characterized by abnormal proliferation of CD1a-positive dendritic cells, which leads to a variety of clinical manifestations.^[1]^ LCH mostly occurs in children, it is considered to be extremely rare in adults with an incidence of 1 to 2 cases every million.^[1]^ LCH may occur in a single organ or multisystem organ diseases, those with multisystem involvement usually have a poor prognosis. Liver involvement occurs in 10.1% to 18% of multisystem LCH in the pediatric patients, which can result in severe complications, including sclerosing cholangitis (SC) with jaundice.^[2]^

Here, we report a 31-year-old patient underwent successful liver transplantation (LT) for severe SC due to multisystem LCH. Interestingly, there is no sign of recurrence of LCH following transplantation for 4 and a half years. The study was approved by our Institutional Review Board, and informed consent was obtained from the patient.

## 2 Case report

**Figure 1:**
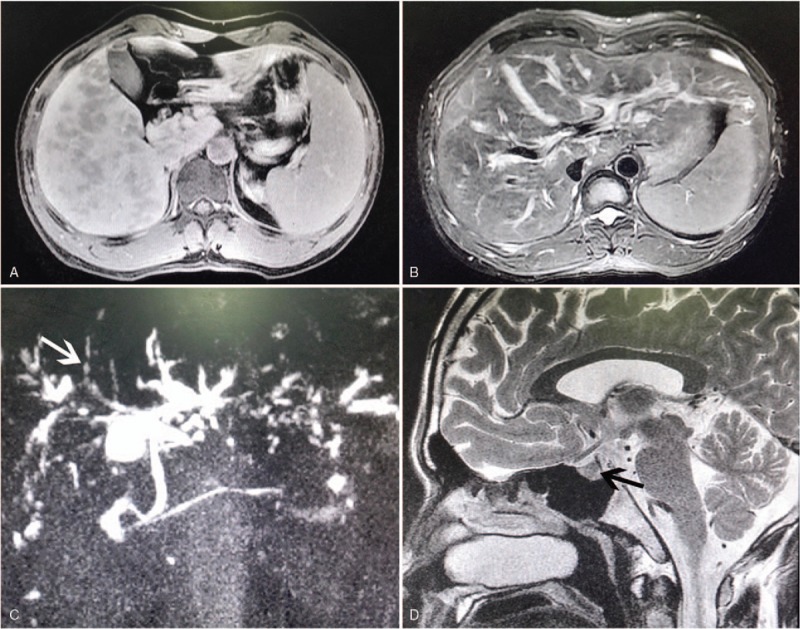
Contrast-enhanced MRI (A and B). (A) The contrast-enhanced T1 signal revealed hypodense nodules scattered in the liver lobes. (B) The dilated intrahepatic bile duct shows high signal intensity, surrounded by the lower-intensity nodules lesions on axial T2-W MR image. (C) MRCP demonstrates sclerosing cholangitis, including segmental intrahepatic bile duct dilatation and stenosis (white arrow). (D) The neurohypophyseal area MRI showed the thickened hypothalamic nuclei and a low-density signal of 4.9 × 5.6 mm in size (black arrow) in the hypothalamic-pituitary area. MRCP = magnetic resonance cholangiopancreatography, MRI = magnetic resonance imaging.

**Figure 2:**
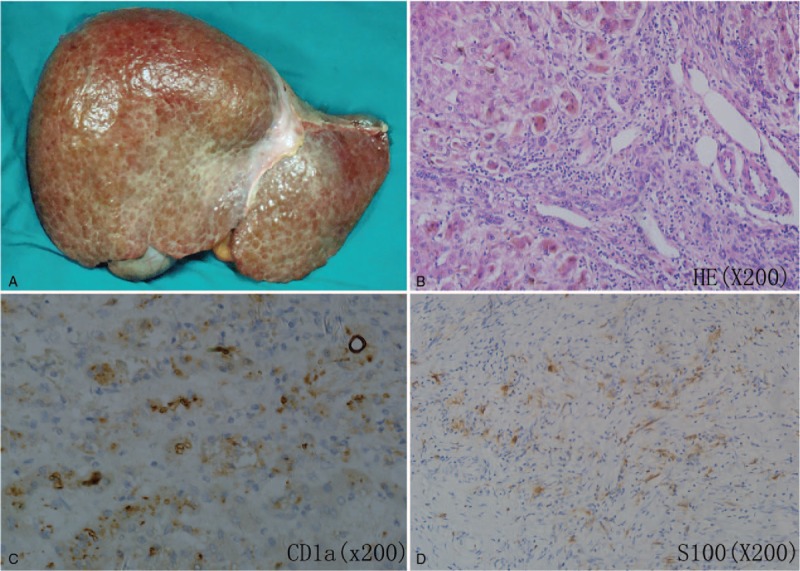
Macroscopic appearance and histopathological findings of liver after liver transplantation. (A) Macroscopic appearance of the liver of the recipient. (B) Shows a massive ductular proliferation, and interstitial fibrosis and inflammatory infiltrates in the portal area. Granuloma with histiocytic infiltration consists of eosinophils and large mononuclear cells with cleaved nuclei (HE × 200). Positive immunohistochemical staining for CD1a (C) and S100 (D) consistent with Langerhans cells (×200).

A 31-year-old man developed diabetes insipidus with urine volume up to 10 to 20 L every 24 hours in 2003. Four years later, he complained of fatigue, anorexia, jaundice and pruritus, and a symptomatic occipital mass. Laboratory tests showed an abnormal liver enzyme (Table 1), the patient was negative for hepatitis viruses. As shown in Fig. 1, abdominal MRI showed multiple low-density lesions in the liver on the T1-weighted image and obvious expansion of the intrahepatic bile duct on the T2-weighted image. Magnetic resonance cholangiopancreatography revealed multifocal intrahepatic bile duct strictures and dilatation, but the common hepatic duct was normal, it was highly suggestive of SC. The neurohypophyseal area MRI showed the thickened hypothalamic nuclei and a low-density signal of 4.9 × 5.6 mm in size in the hypothalamic-pituitary area. A multisystem, high-risk organ LCH was confirmed after occipital mass was biopsied in the local hospital, the patient was given ursodeoxycholic acid 150 mg 3 times a day. Then, he began to receive a course of COEP chemotherapy (cyclophosphamide, 1000 mg; vincristine, 2 mg; epirubicin, 90 mg; and prednisone, 90 mg) in 2009. However, on the 5th day of the 1st COEP chemotherapy, the patient appeared to severe liver function injury with an obvious increase of serum bilirubin (Table 1). He refused further chemotherapy. One year later, the patient developed liver decompensation with bleeding esophageal varices, ascites, and splenomegaly, and he was referred to LT (Model for End Stage Liver Disease score 17). He underwent successful orthotopic LT in November 2011 in our center, and the donor came from voluntary deceased citizen organ donation in China. Liver histopathology after LT revealed micronodular cirrhosis with SC and positive immunostaining (CD1a and S100), suggestive of LCH involving in the liver (Fig. 2). Postoperatively, the man continued to be immunosuppressed with tacrolimus and mycofenolate mofetil. The patient is currently well with normal liver function and no evidence of recurrence of LCH for 4 and a half years follow-up.

**Table 1:**
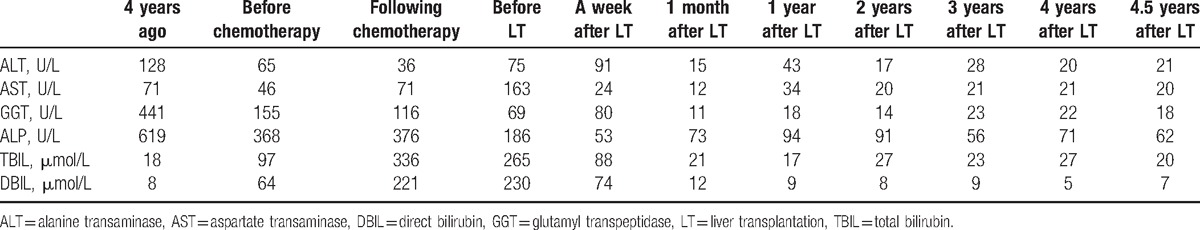
Changes of liver function during different stages.

## 3 Discussion

Liver involvement is well-known in children suffering from multisystem LCH; however, liver LCH is considered to be poorly recognized in adult. Liver involvement in multisystem LCH is regarded as an inferior prognostic indicator.^[2]^ LCH patients with multisystem organ involvement and dysfunction of the liver are considered to be a high-risk group. A definitive diagnosis requires lesional tissue with positive immunohistochemistry staining of CD1a and S100. In this case, he presented with diabetes insipidus, occipital, and liver involvement, with positive immunostaining (CD1a and S100) of liver issues, these finds were consistent with diagnose of multisystem LCH. The histologic examination of the liver LCH demonstrates varying grades of bile duct proliferation, portal tract inflammation, granulomas, fibrohistiocytic proliferation, and portal cirrhosis. In severe patients, the bile duct involvement results in SC that rapidly progresses to cirrhosis and end-stage liver disease, the incidence of SC in multisystem LCH ranges from 10% to 18%.^[2]^ Furthermore, LCH-related SC progresses to biliary cirrhosis more rapidly than primary SC,^[3]^ and have poor response to chemotherapy. Some conventional chemotherapeutic drugs are associated with the development of liver function injury, including vinblastin and cyclophosphamide. Our patient appeared more serious SC due to receiving COEP chemotherapy, with obvious increased serum bilirubin. At present, prednisone and vinblastin is recommended as management of multisystem disease with liver involvement, however, owing to hepatobiliary elimination, vinblastin is contraindicated when cholestasis is present.^[4]^ In additional, cyclophosphamide could result in hepatotoxicity by peroxidative damage to normal liver tissue.^[5]^ Although the real etiology of severe liver function injury in this setting remains unknown, we presume that both vinblastin and cyclophosphamide may be associated with the development of severe liver function injury. Despite with a low incidence, patients receiving chemotherapy including vinblastin and cyclophosphamide are at a high risk of developing severe LCH-related SC complicating cholestasis. Thus, LT should be recommended as an effective treatment for these patients, even if partial chemotherapy response is observed. A recent research showed that a patient's Model for End Stage Liver Disease score reached 15 is regarded as the optimal timing to refer patients for LT, irrespective of etiology.^[6]^ At present, there are only 6 cases of adults with LCH underwent LT that have been published.^[4,7,8]^ We reported a patient with severe SC and was ultimately treated with LT and acquired excellent prognosis. Due to limited experience in rare disease, LT would be an effective therapy for severe SC after LCH in adults, which help us get a better understanding of multisystem LCH and improved diagnosis and treatment.

Considering of the potential autoimmune mechanism of LCH, the immunosuppressive therapy posttransplant could reduce recurrence rate. It has been indicated that cyclosporine might play an important role in the treating and prevention of LCH recurrence.^[8]^ We firstly used tacrolimus and mycofenolate mofetil as immunosuppressants to treat LCH after LT, the patient is currently well with normal liver function and no evidence of recurrence of LCH for 4 and a half years follow-up. The immunological mechanisms of immunosuppressive agents in treating LCH remains unknown, it is possible that immunosuppressants may reduce the reactivation of LCH and SC in the transplanted liver.^[9]^ Herein, only 1 paper showed graft recurrence after 4 years LT in adults, which ultimately treated by chemotherapy.^[10]^

## 4 Conclusion

Our patient presented with severe LCH-related SC and underwent successful LT with the 4 and a half years follow-up, suggesting that LT might be an effective treatment for adults with severe SC due to multisystem LCH. In addition, immunosuppressants as tacrolimus and mycofenolate might be practical to prevent LCH recurrence.
